# 2104. Cytomegalovirus DNAemia Patterns in Mismatched Solid Organ Transplant Recipients: A Retrospective Cohort Study.

**DOI:** 10.1093/ofid/ofac492.1726

**Published:** 2022-12-15

**Authors:** Oscar A Fernandez Garcia, Dima Kabbani, Karen Doucette, Mark Robbins, Carlos Cervera

**Affiliations:** University of Alberta, Edmonton, Alberta, Canada; University of Alberta, Edmonton, Alberta, Canada; University of Alberta, Edmonton, Alberta, Canada; University of Alberta, Edmonton, Alberta, Canada; University of Alberta, Edmonton, Alberta, Canada

## Abstract

**Background:**

Cytomegalovirus (CMV) seronegative solid organ transplant (SOT) recipients who receive grafts from seropositive donors are at high risk of CMV disease. Antiviral prophylaxis in usually given to this group of patients to prevent illness. Patients are still at risk of CMV after completion of prophylaxis. Surveillance after prophylaxis is used in some centres to prevent late CMV disease, but there is scant information of its efficacy. Finally, little is known on CMV kinetics post-prophylaxis in high-risk patients.

**Methods:**

This was a retrospective cohort study including adult CMV mismatch (D+/R-) SOT recipients between 2003-2017 at a multiorgan transplant center in Canada. Post-prophylaxis CMV kinetics were classified into 3 patterns of DNAemia: no DNAemia, single episode of DNAemia and recurrent episodes of DNAemia. We calculated the cumulative incidence of each DNAemia pattern. We also compared 5-year mortality according to CMV DNAemia pattern by Cox-regression analysis. Patients were monitored weekly with CMV viral load for 12 weeks after completion of prophylaxis.

**Results:**

Two-hundred and forty-five transplant patients were included (Table 1). Median follow up time was 9.2 years (7-12.7). Death occurred in 32 patients during the study period, the median time to death was 7.3 years (4.9-10). Pattern 1 (no CMV DNAemia) occurred in 38%, 27% had a single episode (pattern 2) of CMV DNAemia and 35% had recurrent (pattern 3) CMV DNAemia. Median time to DNAemia was 186 days. The first episode occurred at a significantly shorter interval in liver recipients when compared to non-liver recipients, 158 days (134-217) vs. 208 days (149-315), p=0.0164. Recurrent CMV DNAemia (pattern 3) was significantly more common in lung transplant recipients compared to non-lung transplant recipients. (63% vs. 32% p=0.003).

Mortality at 5 years was statistically not different between CMV patterns, adjusted by organ transplanted and age (Pattern 1 reference; pattern 2 HR 1.484 [0.24-9.

**Table 1.**
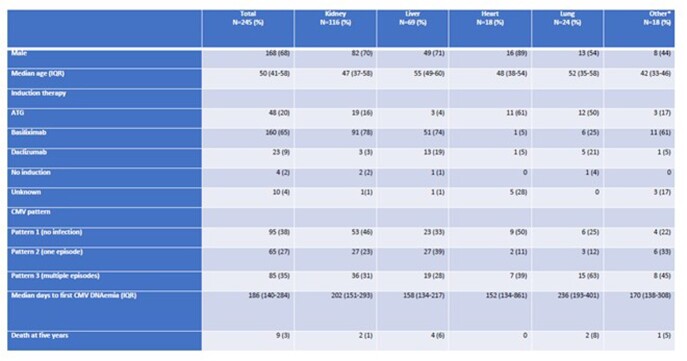
Population characteristics.

**Conclusion:**

A surveillanceafter prophylaxis strategy allowed us to characterize the CMV kinetics post-prophylaxis, with 35% of CMV mismatch patients having recurrent CMV episodes. CMV kinetic pattern was not associated with 5 years mortality in CMV high-risk patients.

**Disclosures:**

**Carlos Cervera, Associate Professor**, Astra-Zeneca: Advisor/Consultant|AVIR Pharma: Grant/Research Support|AVIR Pharma: Honoraria|Lilly: Advisor/Consultant|Merck: Advisor/Consultant|Merck: Grant/Research Support|Merck: Honoraria|Sunovion: Advisor/Consultant|Takeda: Advisor/Consultant|Takeda: Honoraria|VerityPharma: Advisor/Consultant.

**Table 1.** Population characteristics.

* Includes 16 simultaneous kidney-pancreas, pancreas after kidney or pancreas alone, 1 combined liver-kidney, 1 multivisceral. ATG: Anti-thymocyte globulin.

